# Genome Replication Is Associated With Release of Immunogenic DNA Waste

**DOI:** 10.3389/fimmu.2022.880413

**Published:** 2022-05-11

**Authors:** Nadja Schubert, Tina Schumann, Elena Daum, Karolin Flade, Yan Ge, Lara Hagedorn, Winfried Edelmann, Luise Müller, Marc Schmitz, Gunnar Kuut, Veit Hornung, Rayk Behrendt, Axel Roers

**Affiliations:** ^1^ Institute for Immunology, Medical Faculty Carl Gustav Carus, University of Technology (TU) Dresden, Dresden, Germany; ^2^ Department of Cell Biology, Albert Einstein College of Medicine, Bronx, NY, United States; ^3^ National Center for Tumor Diseases (NCT), University Hospital Carl Gustav Carus, University of Technology (TU) Dresden, Dresden, Germany; ^4^ German Cancer Consortium (DKTK), Partner Site Dresden, and German Cancer Research Center (DKFZ), Heidelberg, Germany; ^5^ Gene Center and Department of Biochemistry, Ludwig-Maximilians-Universität München, Munich, Germany; ^6^ Institute for Clinical Chemistry and Clinical Pharmacology, University Hospital Bonn, Bonn, Germany; ^7^ Institute for Immunology, University Hospital Heidelberg, Heidelberg, Germany

**Keywords:** Trex1, type I interferon, Exo1, replication, cytosolic DNA, interferonopathy

## Abstract

Innate DNA sensors detect foreign and endogenous DNA to induce responses to infection and cellular stress or damage. Inappropriate activation by self-DNA triggers severe autoinflammatory conditions, including Aicardi-Goutières syndrome (AGS) that can be caused by defects of the cytosolic DNase 3’repair exonuclease 1 (TREX1). *TREX1* loss-of-function alleles are also associated with systemic lupus erythematosus (SLE). Chronic activation of innate antiviral immunity in TREX1-deficient cells depends on the DNA sensor cGAS, implying that accumulating TREX1 DNA substrates cause the inflammatory pathology. Retrotransposon-derived cDNAs were shown to activate cGAS in TREX1-deficient neuronal cells. We addressed other endogenous sources of cGAS ligands in cells lacking TREX1. We find that induced loss of TREX1 in primary cells induces a rapid IFN response that requires ongoing proliferation. The inflammatory phenotype of *Trex1^-/-^* mice was partially rescued by additional knock out of exonuclease 1, a multifunctional enzyme providing 5’ flap endonuclease activity for Okazaki fragment processing and postreplicative ribonucleotide excision repair. Our data imply genome replication as a source of DNA waste with pathogenic potential that is efficiently degraded by TREX1.

## Introduction

Aberrant activation of innate antiviral immunity is a key pathogenetic principle in a spectrum of autoinflammatory and autoimmune diseases (1–3). Innate sensing of virus infection largely relies on detection of viral nucleic acids. This bears the problem of avoiding sensor activation by endogenous DNA and RNA, a difficult task that the immune system can fail to meet for many different reasons, resulting in chronic expression of type I interferon (IFN) as well as proinflammatory cytokines, and severe, often fatal, inflammatory diseases (1–4). A growing number of distinct human conditions have been reported, currently about 40, that are caused by immune responses to endogenous nucleic acids or other means of inappropriate nucleic acid sensor activation, and are collectively addressed as type I interferonopathies (2, 5).

Double-stranded DNA activates the sensor cGAS in a sequence-independent manner (6). Upon ligand binding, cGAS catalyzes the formation of the second messenger 2’3’-cGAMP which activates STING, resulting in IRF3 and NF-*κ*B activation (7, 8). The cGAS/STING axis is essential for defense against numerous viral and non-viral pathogens (9). However, cGAS does not discriminate between microbial and endogenous DNA and is readily activated by self-DNA (6). Thus, effective safeguard mechanisms are key to avoid uncontrolled activation. cGAS is expressed in the cytosol and in the nucleus (10, 11). Sensor functions of cGAS in the nucleus are incompletely understood. Most nuclear cGAS is tightly tethered to nucleosomes, by binding to the acidic patch formed by nucleosomal core histones and is thereby kept inactive (reviewed in (10, 11). Chromatin structure, nuclear proteins competing with cGAS for the histone acidic patch or DNA, cofactors required for DNA binding, and posttranslational modifications of histones or cGAS, all contribute to regulation of nuclear cGAS DNA sensing (11). Disturbance of histone stoichiometry can result in pathogenic activation of nuclear cGAS by chromosomal DNA (12). Cytosolic cGAS is not exposed to relevant amounts of DNA in healthy, uninfected cells due to efficient cytosolic DNA waste disposal ensured by 3’ repair exonuclease 1 (TREX1, DNase III). This ER-anchored non-processive exonuclease degrades ssDNA, but also dsDNA with overhangs, which it can unwind (13, 14).

*Trex1* is one of several genes, defects of which can each cause the mendelian autoimmune disease Aicardi-Goutières syndrome (AGS) characterized by debilitating encephalitis and inflammation of other organs (15, 16). While homozygous loss of *Trex1* results in AGS, heterozygous *Trex1* loss-of-function alleles are associated with systemic lupus erythematosus (SLE), contributing to the polygenic predisposition for this severe autoimmune disease (17). AGS and SLE overlap clinically and also share key biochemical features, including chronic activation of type I IFN signaling and antinuclear auto-antibodies (18–21).

TREX1-deficient mice reproduce chronic type I IFN response, auto-antibody formation and multi-organ inflammation (22–24), most prominently inflammation of the myocardium, that is fatal by the age of few months, whereas the CNS is only discretely affected (25). *Trex1^-/-^* pathology is largely rescued by additional inactivation of cGAS, STING, IRF3 or IFNAR expression (22, 24, 26). Likewise, IFN expression of human TREX1-deficient cells is abrogated by additional inactivation of the cGAS/STING axis (27). These findings indicate inappropriate cGAS activation by some endogenous TREX1 DNA substrate as the key pathogenetic event.

TREX1 degrades HIV cDNA and limits innate responses to the virus (28). Endogenous retrovirus cDNA can serve as activating ligands for cGAS (29), and de-repression of endogenous retroelements in cells with defective histone methylation is associated with induction of type I IFN signaling (30). TREX1 may serve to continuously degrade replication intermediates of endogenous retroelements (24, 31). While we observed no amelioration of the *Trex1^-/-^* phenotype by treatment with reverse transcriptase inhibitors (32), human TREX1-deficient neurons and astrocytes, however, were shown to accumulate DNA originating from endogenous retroelements in the cytosol and their spontaneous IFN response was inhibited by antiretroviral drugs. Moreover, chronic IFN signaling of AGS patients was ameliorated by treatment with reverse transcriptase inhibitors (27, 33).

Herein, we address other sources of immunostimulatory DNA in cells lacking TREX1. DNA damage can lead to chromosomal aberrations resulting in problems of mitotic chromosome segregation and formation of micronuclei. Collapse of the unstable micronuclear envelope allows cGAS to access micronuclear chromatin. This chain of events triggers the chronic interferon response in AGS caused by defects of RNase H2 (34). Besides retroelement replication intermediates or mislocalized chromatin, the complex metabolism required to ensure genome maintenance, in particular a multitude of repair pathways, but also DNA replication, might contribute activating cGAS ligands. Oligonucleotide DNA waste released during repair and/or replication could stimulate cGAS in TREX1-deficient cells. As oligonucleotides equilibrate between nucleus and cytosol, TREX1-mediated degradation might also reduce nuclear DNA waste and prevent activation of nuclear cGAS (35). We demonstrate that DNA replication is essential for the cGAS/STING response of TREX1-deficient cells and identify exonuclease (Exo)1, an enzyme providing 5’flap-endonuclease activity during Okazaki fragment maturation and postreplicative ribonucleotide excision repair, as an important contributor to the cell-intrinsic innate immune response upon loss of TREX1.

## Results

### Spontaneous DNA Damage, but No Effect of p53-Mediated DNA Damage Responses on Chronic cGAS/STING Activation in *Trex1^-/-^* Mice

**Figure 1:**
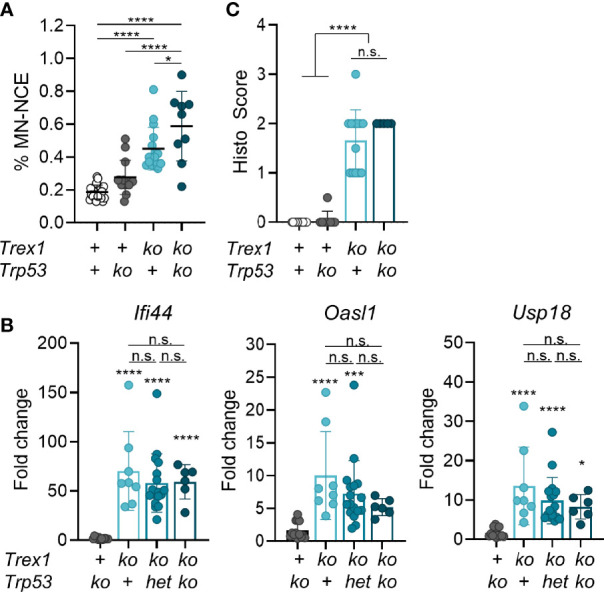
Mild genome damage, but no amelioration of the inflammatory phenotype of *Trex1^-/-^* mice lacking also p53. **(A)** Flow cytometric quantification of micronucleated erythrocytes from 6 week-old mice of the indicated genotypes (WT n = 22, *Trp53* ^-/-^ n = 14, *Trex1* ^-/-^ n = 16, *Trp53* ^-/-^ *Trex1* ^-/-^ n = 9). See **Figure S1** for gating strategy. The *Trex1* null allele published by Morita et al. was used (23). **(B)** ISG transcript levels in total blood cells quantified by qRT-PCR. Fold change compared with mean of WT controls (n = 16) is shown. *Trp53* ^-/-^ n = 13, *Trex1* ^-/-^ n = 8, *Trex1* ^-/-^ *Trp53* ^WT/-^ n = 17, *Trex1* ^-/-^ *Trp53* ^-/-^ n = 6. **(C)** Quantification of myocarditis intensity in H&E-stained sections of heart tissue from 6-10 week-old mice of the indicated genotypes (WT n = 11, *Trp53^-/-^* n = 9, *Trex1^-/-^* n = 12, *Trex1^-/-^Trp53^-/-^* n = 5; see *M&M* for heart inflammation score). ****p < 0.0001, ***p < 0.001, *p < 0.05, n.s., not significant.

*Trex1^-/-^* cells feature tonic DNA damage checkpoint signaling (36), increased numbers of strand breaks and spontaneous phosphorylation of p53 (35). In AGS caused by defects of RNase H2 (37), extensive genome damage results in micronucleus formation which drives the chronic IFN response of these patients (34). This response is counter-regulated by p53-dependent DNA damage responses which eliminate cells with high damage load and prevent them from prolonged IFN production (38). Micronuclear DNA or chromatin bridges were shown to trigger an IFN response in cancer cells treated with genotoxic drugs (39, 40), and TREX1 was reported to limit cGAS activation in genetically unstable cells by degradation of micronuclear DNA (41). Micronucleated cells can be quantified by flow cytometric analysis of erythrocytes, as erythrocyte precursors expel the main nucleus but frequently retain micronuclei (42). We found increased numbers of micronucleated erythrocytes in *Trex1^-/-^* mice (**Figure 1A** and **Figure S1**), reflecting spontaneous DNA damage and chromosomal aberrations. However, compared to a 30-fold increase in mice lacking RNase H2 in the hematopoietic system (manuscript in preparation), micronucleated cells were only mildly (2-fold) increased in *Trex1^-/-^* mice. To address whether cells carrying genome damage are an important source of type I IFN in *Trex1^-/-^* mice, we crossed *Trex1^-/-^* to *Trp53^-/-^* animals (43) and compared the IFN response of double knock out mice to that of *Trex1^-/-^* littermates. Additional loss of p53 further increased fractions of micronucleated erythrocytes (**Figure 1A**), but did not increase transcript levels of type I IFN-stimulated genes (ISGs) in total blood cells (**Figure 1B**) nor enhance inflammation of the myocardium in TREX1-deficient mice (**Figure 1C**). These data strongly argue against genome damage and cytosolic chromatin as the trigger of cGAS activation in the absence of TREX1 and lead us to investigate alternative sources of TREX1-sensitive cGAS activating DNA ligands.

### Neither Nucleotide Excision Repair Nor the Structure-Specific Endonuclease MUS81 Are a Relevant Source of Immunostimulatory Oligonucleotide Waste Accumulating in *Trex1^-/-^* Cells

**Figure 2:**
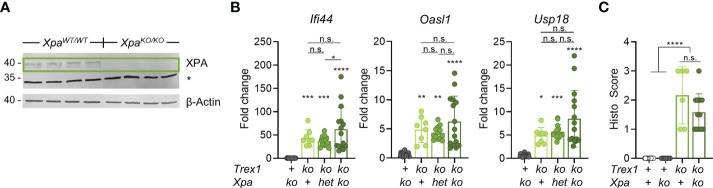
Additional inactivation of NER has no impact on the spontaneous IFN response of *Trex1^-/-^* mice. **(A)** Western blot analysis of XPA expression in protein extracts from ear tissue from control and *XPA^-/-^* mice. * non-specific band at 34 kDa. **(B)** ISG transcript levels in total blood cells from 6 week-old mice of the indicated genotypes quantified by qRT-PCR. The *Trex1^Δ478^* null allele generated in this study was used. Fold change compared with mean of WT controls (n = 16) is shown (*Trex1^-/-^* n = 8, *Xpa^-/-^* n = 18, *Trex1^-/-^Xpa^WT/-^* n = 13, *Trex1^-/-^Xpa^-/-^* n = 15. **(C)** Quantification of myocarditis intensity in H&E-stained sections of heart tissue from 9-11 week-old mice of the indicated genotypes (WT n = 15, *Xpa^-/-^* n = 7, *Trex1^-/-^* n = 6, *Trex1^-/-^Xpa^-/-^* n = 12). See *M&M* for heart inflammation score. ****p < 0.0001, ***p < 0.001, **p < 0.01, *p < 0.05, n.s., not significant.

Primary TREX1-deficient fibroblasts from AGS patients not exposed to experimental genotoxic hazards (35, 36) showed DNA staining in the cytosol, suggesting leakage of DNA from the nucleus or mitochondria in healthy cells. Higher amounts of DNA seem to leak into the cytosol of genetically unstable cancer cells, in particular after exposure to genotoxic hazards (44–48). This DNA, likely a waste from DNA repair (44), seems to overwhelm TREX1 exonuclease activity (44, 48). Whether the same mechanisms generating large amounts of TREX1 substrates in genome-damaged cells also contribute to release of DNA from the nucleus in healthy cells is unknown. Several DNA repair pathways involve release of oligonucleotides that could, upon hybridization, form partially double-stranded structures, e.g. Y-DNA (29), capable of ligating cGAS. Nucleotide excision repair (NER) removes bulky DNA lesions by excision of short single-stranded patches containing the lesions (49). High amounts of these, 22-30 nt-long flaps may be released also in the absence of exogenous genotoxic stress, since NER is triggered already by minimal distortions of undamaged DNA resulting in low-level DNA turnover in healthy cells termed ‘gratuitous repair’ (50, 51). Moreover, we had earlier found that in contrast to cell-intrinsic activation of transcriptional responses to loss of TREX1 in several different cell types (25), this study and unpublished), inactivation of TREX1 in B cells had no detectable effect on the transcriptome of murine B cells (25). Since B cells were reported to be NER-deficient (52), our observation would be plausibly explained by NER being a source of the cGAS ligand accumulating in *Trex1^-/-^* mice. We therefore generated mice double-deficient for TREX1 and XPA, an essential NER protein (49, 53), using CRISPR/Cas9-mediated mutagenesis in embryos (**Figure S2A**). As expected, *Xpa^-/-^* animals (**Figure 2A**) showed a loss of repair synthesis in UV-irradiated cells (**Figure S2B**), indicating absence of NER function. We compared ISG expression in blood cells of mice lacking TREX1 or XPA and of *Trex1^-/-^Xpa^-/-^* animals (**Figure 2B**). *Xpa^-/-^* mice proficient for TREX1 did not show enhanced ISG expression compared to control mice. IFN signaling was not reduced in the double mutants compared to *Trex1^-/-^* mice. Moreover, the myocarditis typical of TREX1-deficient mice was not ameliorated by additional inactivation of NER (**Figure 2C**).

**Figure 3:**
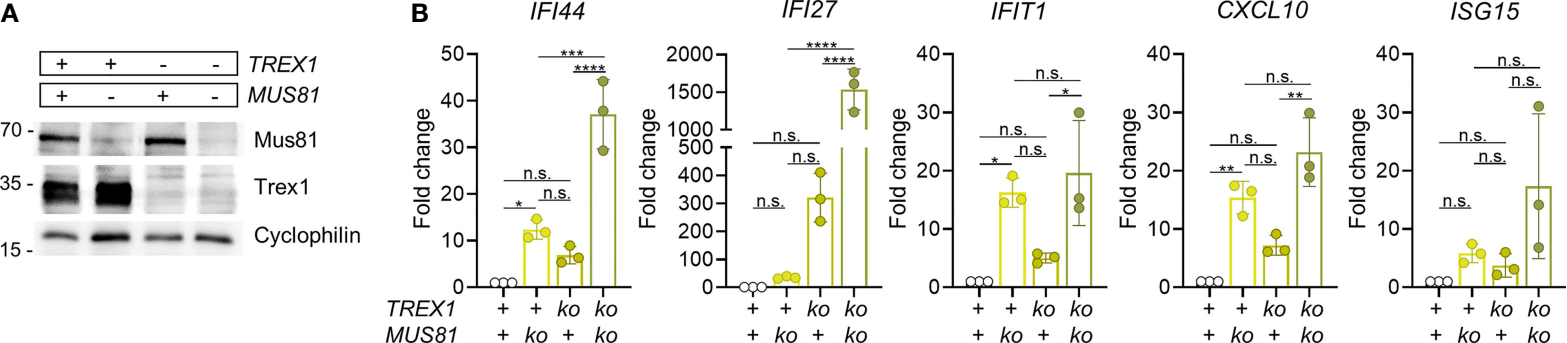
Simultaneous inactivation of *Trex1* and of *Mus81* result in synergistically enhanced IFN response. **(A)** Western blot analysis of MUS81 expression by polyclonal cultures resulting from lentiviral transduction of control or *Trex1^-/-^* THP1 cells (see **Figure S3**) with lentiCRISPR v2 plasmid encoding *Mus81*-specific guide RNA (TCTGAAATACGAAGCGCGTG) and selection for the presence of the plasmid. **(B)** ISG transcript levels in THP1 cells of the indicated genotypes quantified by qRT-PCR. Fold change compared with mean of WT controls is shown. Graph summarizes 3 independent experiments. ****p < 0.0001, ***p < 0.001, **p < 0.01, *p < 0.05, n.s., not significant.

MUS81 is a DNA repair factor reported to be a source of cytoplasmic DNA in cancer cells (54). This structure-specific endonuclease is essential for genome integrity as it is required for resolution of Holiday junctions (55) and prevention of chromosome bridges in anaphase (56), as well as repair of stalled and collapsed replication forks (57). MUS81 function has important impact on the activity of the type I IFN system [reviewed in (58)]. Loss of the enzyme results in pronounced spontaneous IFN production (59). As MUS81 deficiency leads to chromosomal instability, a likely scenario is that micronuclear DNA is the key stimulator of this IFN response. Due to this spontaneous IFN response of MUS81-deficient cells, the question whether MUS81 contributes to production of the immunostimulatory cGAS ligand accumulating in otherwise undamaged cells in the absence of TREX1, cannot be directly addressed by inactivation of MUS81 in *Trex1^-/-^* cells or mice. However, in case the IFN response of cells lacking TREX1 depended on DNA waste produced by MUS81, no synergistic IFN induction by TREX1 deficiency and MUS81 deficiency would be expected. We inactivated TREX1 and/or MUS81 in THP1 cells (**Figure 3A**). The robust spontaneous ISG expression of *TREX1^-/-^* cells was rescued by reconstitution with functional TREX1 (Fig S3A&B). As expected, also *MUS81^-/-^* cells featured prominent type I IFN signaling (**Figure 3B**). The double deficient cells, however, showed synergistically enhanced ISG expression (**Figure 3B**). This result argues that MUS81 is not required for generation of the cGAS ligand accumulating as a result of TREX1 deficiency. Collectively, our data rule out NER as a dominant source of the endogenous immunostimulatory DNA accumulating in mouse cells lacking TREX1 and argue against a significant contribution of the structure-specific endonuclease MUS81 to generation of this pathogenic cGAS ligand.

### Partial Rescue of the *Trex1* ^-/-^ Phenotype by Additional Inactivation of Exonuclease 1

**Figure 4:**
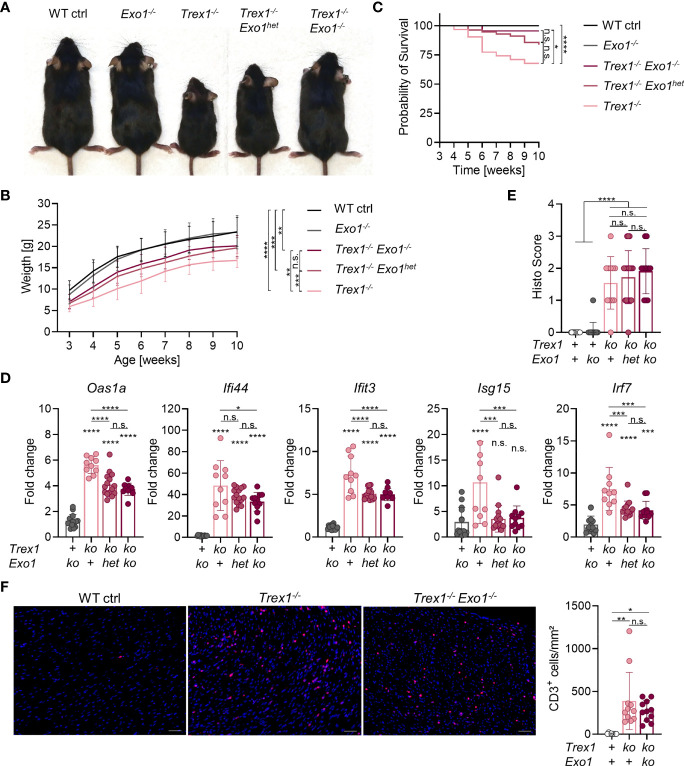
Partial rescue of the Trex1 inflammatory phenotype by additional inactivation of *Exo1*. **(A)** Macroscopic phenotype of 7-8 week-old mice of the indicated genotype. **(B)** Body weight of animals of the indicated genotype (WT n = 32, *Exo1^-/-^* n = 20, *Trex1^-/-^* n = 26, *Trex1^-/-^Exo1^+/-^* n = 46, *Trex1^-/-^Exo1^-/-^* n = 15). Statistical analysis was performed for the comparison of body weight at the age of 10 weeks. **(C)** Survival of mice of the indicated genotype until the age of 10 weeks (WT n = 46, *Exo1^-/-^* n = 31, *Trex1^-/-^* n = 31, *Trex1^-/-^Exo1^+/-^* n = 56, *Trex1^-/-^Exo1^-/-^* n = 22). **(D)** ISG transcript levels in total blood cells from 6 week-old mice of the indicated genotypes quantified by qRT-PCR. Fold change compared with mean of WT controls (n = 15) is shown (*Trex1^-/-^* n = 10, *Exo1^-/-^* n = 14, *Trex1^-/-^Exo1^WT/-^* n = 15, *Trex1^-/-^Exo1^-/-^* n = 11). See **Figure S4** for expression of additional ISGs. **(E)** Quantification of inflammatory infiltration on H&E-stained sections of heart tissue from 10-12 week-old mice of the indicated genotypes. **(F)** Immunohistochemical staining of CD3 on sections of heart tissue from 10-12 week-old mice of the indicated genotypes (left). Scale bar 50 µm. Quantification of CD3^+^ cells per area of heart muscle by investigator-blinded automated image analysis (right). ****p < 0.0001, ***p < 0.001, **p < 0.01, *p < 0.05, n.s., not significant.

We next turned our attention to another repair factor that had been shown to contribute to release of TREX1-sensitive DNA into the cytosol of genome-damaged cells (44), exonuclease 1 (EXO1). Homology-directed repair of double-strand breaks requires long-range resection of the 5’-end in order to produce the long single-stranded 3’-overhangs that are the substrates for strand invasion (60). EXO1 is a multifunctional enzyme with 5’-3’ exonuclease activity, but also 5’-flap structure-specific endonuclease activity, involved in end processing in preparation for homology-directed repair as well as in telomere homeostasis, mismatch repair, and Okazaki fragment maturation during lagging strand synthesis (61). Upon exposure of cells to genotoxic irradiation or drugs inducing double-strand breaks, 5’-end resection, performed by EXO1 and other endonucleases, was shown to be a source of DNA waste triggering a type I IFN response (44). TREX1-mediated degradation limited the innate immune response (44). We therefore asked, whether end resection by the EXO1 endonuclease activity might yield DNA waste also during steady-state genome repair. In addition, the EXO1 5’-flap cleavage occurring during genome replication might also release immunostimulatory DNA waste. We crossed *Exo1^-/-^* mice (62) to TREX1-deficient mice and compared littermates of different genotypes for weight gain and spontaneous type I IFN response. The conspicuous failure to thrive typical of *Trex1^-/-^* mice was largely, albeit not completely, rescued by the additional inactivation of EXO1 (**Figures 4A, B**). Even heterozygosity for the *Exo1* knock out allele resulted in a significant rescue of *Trex1^-/-^* weight gain. Survival of *Trex1^-/-^* mice was significantly improved by additional loss of EXO1 (**Figure 4C**). Correspondingly, type I IFN signaling was clearly reduced in *Trex1^-/-^* mice lacking functional *Exo1*, as determined by quantification of ISG transcript levels in blood cells (**Figure 4D** and **Figure S4A**). For most ISGs, the increase in transcript levels seen in *Trex1^-/-^* mice was blunted to about half in *Trex1^-/-^Exo1^-/-^* animals (**Figure 4D** and **Figure S4A**). Type I IFN bioactivity was not elevated above the background of control animals in serum of *Trex1^-/-^* or *Trex1^-/-^Exo1^-/-^* mice (**Figure S4B**). A control experiment demonstrated that STING responses of EXO1-deficient cells are of normal intensity (**Figure S4C**). The reduced innate response was not reflected in amelioration of myocardial inflammation in the double vs. the *Trex1* single mutants as assessed by histological analysis of inflammatory infiltration on H&E- (**Figure 4E**), anti-CD3- (**Figure 4F**) and van Gieson-stained sections (not shown). Collectively, we show that additional loss of EXO1 significantly ameliorates the spontaneous innate antiviral response of *Trex1^-/-^* animals, suggesting a role for EXO1 endonuclease activity in generation of cGAS-activating ligands in TREX1-deficient cells.

### Rapid Onset of Type I IFN and NF-κB Responses Upon Induced Inactivation of TREX1 in Primary Cells

**Figure 5:**
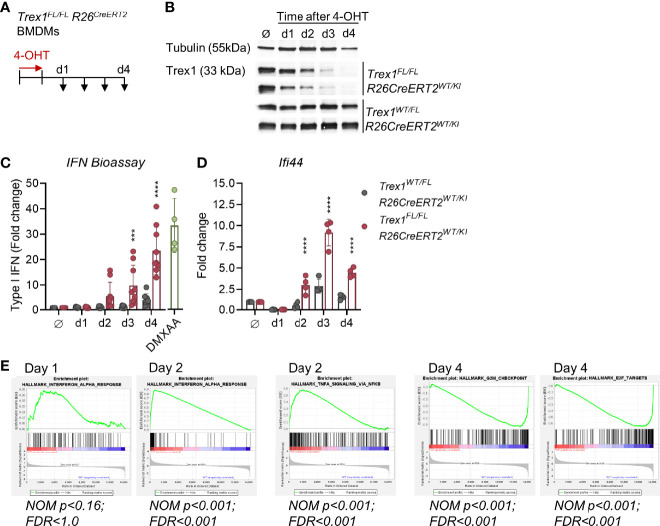
Rapid loss of TREX1 and onset of type I IFN responses upon induced inactivation of *Trex1 in vitro*. **(A)** Inactivation of the *Trex1* gene was induced in macrophages differentiated from *Trex1^FL/FL^R26^CreERT2^* bone marrow (BMDMs) by culture in the presence of 4-OHT for 1 day. 4-OHT treated BMDMs from *Trex1^WT/FL^R26^CreERT2^* mice as well as non-4-OHT-treated cells from both genotypes were used as controls. Samples were obtained on days 1-4 after end of 4-OHT induction. **(B)** Western blot analysis of TREX1 expression in BMDMs at the indicated time points after 4-OHT induction, representative of 6 animals per genotype. **(C)** Assay for type I IFN bioactivity in supernatant of BMDMs sampled at the indicated time points after 4-OHT induction. Supernatant was added to 1x10^4^ LL171 luciferase IFN-reporter cells followed by quantification of luciferase activity (*Trex1^FL/FL^R26^CreERT2^* n = 9, control n = 8). Supernatant of WT BMDMs treated with DMXAA served as high control. **(D)** Transcript levels of the ISG *Ifi44* determined by qRT-PCR in BMDMs sampled at the indicated time points after 4-OHT induction. Fold change compared to untreated control cells (n = 4) is shown. **(E)** Gene set enrichment analysis of RNA sequencing data from *Trex1^FL/FL^R26^CreERT2^ *vs. control (*Trex1^WT/FL^ R26^CreERT2^*) BMDMs sampled at the indicated time points after 4-OHT induction. ****p < 0.0001, ***p < 0.001.

To elucidate the contribution of EXO1 to generation of TREX1 substrates, we first established an *in vitro* model of induced loss of TREX1 expression. We differentiated macrophages from bone marrow (BMDMs, **Figure S5A**) of *Trex1^FL/FL^ R26^CreERT2^* mice (25) and induced Cre activity in *Trex1^FL/FL^ R26^CreERT2^* BMDMs by 4-hydroxytamoxifen (4-OHT, **Figure 5A**). This resulted in efficient deletion of the loxP-flanked *Trex1* allele one day after the end of 4-OHT exposure, as determined by PCR (**Figure S5B**). Western blot analysis of cells harvested at daily intervals after 4-OHT induction (**Figure 5A**) revealed rapid decline of TREX1 protein which was almost completely lost 3 days after the end of induction and was undetectable on day 4 (**Figure 5B** and **Figure S5C**). Increased type I IFN bioactivity was detectable (not significant) in the culture supernatant already on day 2 after end of induction, but was very prominently increased on days 3 and 4 (**Figure 5C**). Transcript levels of the ISG *Ifi44* were significantly increased already on day 2 after 4-OHT and were 10-fold up-regulated on day 3 (**Figure 5D**). RNA sequencing of 4-OHT-induced BMDMs harvested on days 1 to 4 revealed robust up-regulation of ISGs (**Figure 5E** and **Figures S5D, E**). Enrichment of the gene set ‘IFNα response’ was not significant on day 1 after end of 4-OHT, but very much so on days 2 to 4 (**Figure 5E** and **Figures S5D, E**). In addition to ISG induction, loss of TREX1 was reflected in up-regulation of NF-κB and TNF-induced genes on day 2, as expected for STING responses (**Figure 5E**). Gene signatures associated with cell cycle progression, including e.g. expression of proliferation marker MKI67 were significantly downregulated, most prominently on day 4, while expression of CDKN1A encoding cell cycle inhibitor p21 was upregulated (not shown), most likely reflecting IFN-induced cell cycle arrest (**Figure 5E**). Similar results were obtained in *Trex1^FL/FL^ R26^CreERT2^* embryonic fibroblasts (MEFs) (**Figure S5F**).

Collectively, our finding that the IFN response is detectable already at day 2 after 4-OHT induction, a time point at which large amounts of TREX1 were still present, indicates that even slight reductions of TREX1 expression levels result in accumulation of the cGAS ligand and activation of cGAS/STING responses. Thus, steady-state TREX1 expression levels of healthy cells are just sufficient to degrade its DNA substrate. This finding is compatible with ongoing large-scale production of this substrate by at least a fraction of the cells in the culture at any time point.

### The Type I IFN Response of TREX1-Deficient Cells Requires Ongoing Proliferation

Large amounts of oligonucleotide waste might originate from genome replication. Millions of Okazaki fragments are precisely ligated during lagging strand synthesis. Okazaki fragment synthesis by Polδ continues beyond the 5’-end of the previous Okazaki fragment, displacing a 5’-flap that requires endonucleolytic trimming (63). Nick translation by iterative rounds of strand displacement synthesis and flap cleavage not only remove the RNA primer but also the 5’-portion of primer initiator DNA of the previous Okazaki fragment synthesized by POLα. In addition to FEN1, the major flap endonuclease involved in Okazaki fragment maturation, also DNA2 and EXO1 contribute. A recent study suggested that primarily FEN1 and EXO1 compete for Okazaki fragment flap cleavage, with DNA2 playing only a minor role (64). EXO1 overexpression in yeast can partially compensate loss of FEN1 (65).

**Figure 6:**
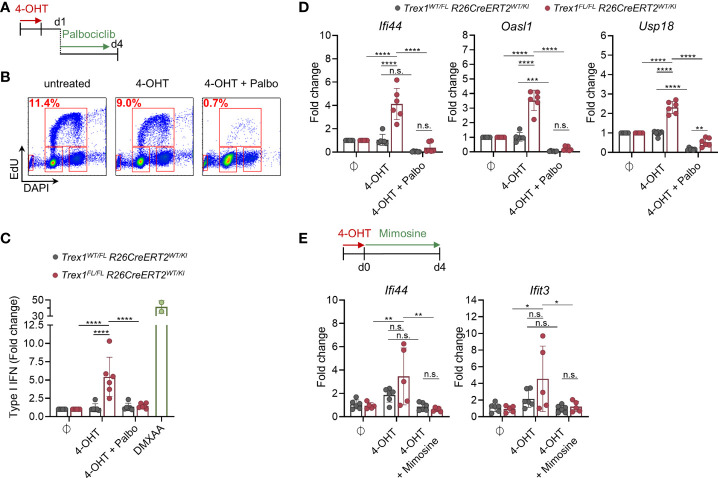
IFN response upon induced loss of TREX1 in macrophage cultures requires ongoing proliferation. **(A)** *Trex1^FL/FL^R26^CreERT2^* and control BMDMs were treated with 4-OHT *in vitro* for 22h. 1 day after end of 4-OHT induction, CDK4/6 inhibitor Palbociclib was added to arrest the cells in G1. **(B)** Verification of proliferative arrest after 72h of Palbociclib treatment (d4 after end of 4-OHT) as determined by flow cytometric quantification of EdU incorporation and DNA content (DAPI). Representative of 3 independent experiments. **(C)** Quantification of type I IFN bioactivity in supernatant from BMDMs treated with 4-OHT and Palbociclib (n = 6 each genotype, pooled from 3 independent experiments). Supernatant was added to 1x10^4^ LL171 luciferase IFN-reporter cells followed by quantification of luciferase activity. Supernatant of WT BMDMs treated with DMXAA served as high control. **(D)** ISG transcript levels in BMDMs after 72h of Palbociclib treatment (d4 after end of 4-OHT) as determined by qRT-PCR (n = 6 for each genotype, pooled from 3 independent experiments). Fold change compared to mean of untreated control cells (n = 6) is shown. **(E)** *Trex1^FL/FL^R26^CreERT2^* and control BMDMs were treated with 4-OHT *in vitro* for 22h. At the end of 4-OHT induction, Mimosine was added to arrest the cells in G1. ISG transcript levels in MEFs after 96h of Mimosine treatment (d4 after end of 4-OHT) as determined by qRT-PCR (*Trex1^WT/FL^R26^CreERT2^* n = 6, *Trex1^FL/FL^R26^CreERT2^* n = 5, pooled from 2 independent experiments). ****p < 0.0001, ***p < 0.001, **p < 0.01, *p < 0.05, n.s., not significant.

Whether DNA waste originating from Okazaki fragment maturation can trigger cGAS/STING responses is unknown. To approach this question, we asked whether the IFN response of cells lacking TREX1 requires DNA replication. This is not easily addressed by experiments aiming to arrest the cell cycle of constitutively TREX1-deficient cells, because immunostimulatory cGAS ligands, formed before halting proliferation, may be stable in the absence of TREX1 and maintain the IFN response. We therefore induced inactivation of *Trex1* in cells *in vitro* and simultaneously arrested the cell cycle. Treatment of BMDM cultures with the CDK4/6 blocker Palbociclib, arresting cells in G1 (66), for 3 days, reduced the fraction of cells in S-phase 10-fold (**Figures 6A, B**). Palbociclib administration in parallel to 4-OHT-induced knock out of *Trex1* (**Figure S6A**) abrogated the IFN response triggered by loss of TREX1, as determined by quantification of type I IFN bioactivity in culture supernatant (**Figure 6C**) and ISG transcript levels (**Figure 6D**). A control experiment demonstrated that BMDMs arrested by Palbociclib are capable of mounting cGAS/STING responses (**Figure S6B**). To independently confirm that the IFN response to induced loss of TREX1 depends on proliferation, we repeated the experiment in a different cell type, MEFs derived from *Trex1^FL/FL^ R26^CreERT2^* mice, using a different cell cycle inhibitor, Mimosine, that is chemically unrelated to Palbociclib and arrests cells in late G1 by activating checkpoint signaling independent of DNA damage (67). 4-OHT-induced inactivation of *Trex1* resulted in an IFN response that was blunted by Mimosine-mediated G1 arrest (**Figure 6E** and **Figure S6C**). A control experiment demonstrated unimpaired IFN responses of Mimosine-exposed cells to STING activation by DMXAA (**Figure S6C**). Collectively, the IFN response of TREX1-deficient cells requires ongoing proliferation and functional EXO1, compatible with oligonucleotides released by flap endonucleases during lagging strand synthesis as the pathogenic ligand for cGAS.

## Discussion

The concept that endogenous DNA waste poses a major threat to immune homoeostasis, can potentially trigger lethal disease, and is therefore continuously eliminated by efficient DNA waste disposal in healthy mammals, was fueled by the discovery that defects of DNases in different compartments cause autoinflammatory nucleic acid sensor activation associated with chronic type I IFN responses and consecutive autoimmunity. Defects of the endonuclease DNase I that degrades chromatin debris in the circulation and other extracellular compartments result in Lupus-like disease in mice (68, 69) and low serum DNase I activity is associated with SLE (70, 71). Lysosomal DNase II is key to degrade chromatin in macrophages that phagocytose nuclei expelled by erythrocyte precursors and to clearance of damaged nuclear DNA by autophagy (72). Loss of this enzyme triggers IFN-driven disease in mouse and man (73, 74) Likewise, mutations compromising removal of dead cells and efficient disposal of their chromatin, including e.g. defects of the complement system, are linked to IFN-driven pathology (75). AGS caused by *TREX1* mutations was paradigmatic for monogenic autoinflammatory and autoimmune conditions resulting from uncontrolled nucleic acid sensor activation and inspired coining of the term ‘interferonopathies’ (2). The observation that defects of the cytosolic exonuclease TREX1 cause monogenic cGAS/STING-driven AGS and are associated with SLE extended the concept of DNA waste disposal to the cytosolic and/or nuclear compartments and raised questions about nature and origin of the immunogenic TREX1 DNA substrates that trigger disease in the absence of the nuclease.

We show that the spontaneous type I IFN response of *Trex1* knock out mice is reduced by additional absence of functional EXO1 and that IFN production of TREX1-deficient cells *in vitro* required ongoing proliferation. These findings are compatible with oligonucleotide waste generated by the flap endonucleases during lagging strand Okazaki fragment maturation as an important source of the pathogenic cGAS ligand accumulating in TREX1-deficient cells. In contrast to long Okazaki fragments in prokaryotes, mammalian Okazaki fragments are only 150-200 nt in length (76), and about 10^7^ fragments are processed per S phase in mammalian cells. Maturation of each fragment involves displacement of its 5’-RNA end by polymerase δ elongating the next fragment. Strand displacement synthesis usually halts after displacement of only one nucleotide followed by cleavage of this one-nucleotide 5’-flap by flap endonuclease FEN1. Iterative cycles of strand displacement and flap cleavage result in removal of the Okazaki fragment RNA primer and the subsequent DNA sequence that was synthesized by error-prone polymerase α. This ‘nick translation’ maintains a ligatable nick that is eventually sealed by DNA ligase I. In A/T-rich sequences, much longer 5’-flaps can be displaced which are rapidly covered by RPA and are no longer substrates for FEN1 (63). These long flaps are cleaved by the flap endonuclease DNA2 in yeast (63). This ‘two nuclease model’ of Okazaki fragment processing was recently extended to include a contribution by EXO1 flap endonuclease activity. Overexpression of EXO1 can compensate for a loss of FEN1 (RAD27) or DNA2 in yeast (65). RAD27 and EXO1 were shown to redundantly process lagging strand flaps and the inactivation of the *Rad27* or the *Exo1* gene reduced strand displacement synthesis by Polδ (64).

Upon accumulation in large amounts in the absence of TREX1, long flaps originating from lagging strand synthesis are likely to form cGAS ligands by random hybridization, considering that 2/3 of mammalian genomes are comprised of repetitive sequence, including tandem repeats and transposable elements (77, 78). Moreover, the capacity of single strand-binding proteins that might prevent hybridization was shown to be exhausted in TREX1-deficient cells (35). Hybridization of oligonucleotides will not efficiently form higher order structures promoting cGAS activation by phase separation (79). However, random hybridization will frequently yield Y-DNA structures, i.e. double-stranded DNA with unpaired ends, which were shown to activate cGAS, provided unpaired guanosines are contained in the single-stranded overhangs (29). Flaps originating from Okazaki fragment maturation can include the Okazaki fragment RNA primer and form RNA:DNA hybrids upon hybridization, which were shown to be suboptimal cGAS ligands, but do activate the sensor (80). The role for EXO1 in generation of an immunogenic waste of flaps during lagging strand replication could be either in actual cleavage of long flaps, but also in ensuring strand displacement synthesis to proceed, thereby allowing generation of long flap structures. In yeast, loss of EXO1 or FEN1 resulted in reduced strand displacement synthesis (64). In line with a concept of DNA replication as a source of immunogenic DNA waste, Yang et al. observed cytosolic accumulations of BrdU-labeled single-stranded oligonucleotides in BrdU-pulsed TREX1-deficient fibroblasts (36).

Of note, replication-dependent and EXO1-dependent generation of immunogenic oligonucleotide waste on a large scale might occur not only during lagging strand synthesis but also during postreplicative ribonucleotide excision repair (81). Millions of single ribonucleotides are incorporated into the genomic DNA during each S phase and their rapid removal involves incision by RNase H2 followed by strand displacement synthesis and flap cleavage by FEN1 and EXO1 (81). EXO1 also contributes to other repair pathways, including repair of double-strand breaks by homologous recombination repair (HRR). In this pathway EXO1 is involved in end resection in preparation for strand invasion. While end resection was shown to be a source of DNA accumulating in the cytosol of genetically unstable tumor cells and even more so after additional experimental genome damage (44), we posit that steady state repair of double-strand breaks in genetically stable cells not exposed to genotoxic hazards does not occur on a large scale and therefore does not yield oligonucleotide waste in amounts sufficient to form cGAS ligands by random hybridization. In contrast, flap cleavage occurs on a massive scale during genome replication.

While our findings strongly suggest Okazaki fragment processing and/or postreplicative repair as an important source of immunogenic DNA waste in mice, Thomas et al. demonstrated that LINE1 retrotransposons are a major source of cytosolic DNA in human TREX1-deficient neuronal cells (27). Neurons and astrocytes differentiated from pluripotent stem cell lines rendered TREX1-deficient by CRISPR-mediated targeted mutagenesis, accumulated LINE1-derived, reverse transcribed DNA in their cytosol and mounted IFN responses that were inhibited by reverse transcriptase inhibitors (27). Type I IFN produced by astrocytes exerted toxic effects on neurons (27). In line with this observation, Rice et al. reduced the IFN and ISG expression of AGS patients by treatment with antiretroviral drugs (33). Both, replication-derived DNA waste and retroelement-derived cDNA may contribute to cGAS activation in TREX1-deficient mammals *in vivo*, with differential relevance in different species and cell types. Replication-derived waste plausibly plays no role in postmitotic neurons and astrocytes. Transposable elements evolved to be expressed and mobile in embryonic tissues, particularly in the germ-track, but to be largely inactive in somatic cells (82). However, as exceptions to this principle, some somatic tissues, including the CNS, show prominent transposon activity, likely reflecting an advantage to organismal function by somatic genetic diversification (83, 84). Transposable element activity also differs significantly between mouse and man (85). In addition to efficient reduction of IFN responses in the CNS as determined by IFNα levels in cerebrospinal fluid, antiretroviral treatment also reduced serum IFN and IFN signaling in blood cells (33). Whether this amelioration of IFN responses outside the CNS might partially be due to blockade of P2X7-mediated caspase activation and reduction of inflammatory cell death by the nucleoside RTIs (86) is currently unclear.

Genome damage and chromosomal translocations trigger cGAS/STING responses by micronuclear chromatin. TREX1-deficient cells exhibit mild genome damage and TREX1 was shown to contribute to degradation of micronuclear DNA (41), in line with the slight increase in numbers of micronucleated cells we observed in *Trex1^-/-^* mice. If micronuclear DNA was an important cGAS ligand in TREX1-deficient cells, however, IFN signaling would be expected to increase upon additional loss of p53, whereby cells carrying severe genome damage would be allowed to survive longer and continue to produce IFN. As IFN signaling was not enhanced in *Trex1^-/-^Trp53^-/-^* mice compared to p53-proficient *Trex1^-/-^* animals, we conclude that TREX1-mediated elimination of micronuclear chromatin and control of STING activation by micronuclei or chromatin bridges is relevant in genetically unstable, genome-damaged cells, rather than in TREX1-deficient, but otherwise healthy, primary cells.

Collectively, we demonstrate that activation of innate antiviral immunity of TREX1-deficient cells depends on their active proliferation and that additional loss of EXO1 significantly reduces the type I IFN response. While hyperactivity of EXO1 exonuclease activity in DNA mismatch-repair-deficient cancer cells was shown to activate the cGAS/STING pathway by induction of genome instability and consecutive mislocalization of chromatin to the cytosol (87), our findings strongly suggest 5’-flap cleavage during Okazaki fragment processing and/or during postreplicative ribonucleotide excision repair as the source of the pathogenic cGAS ligand accumulating in the absence of TREX1. We propose that genome replication of mammalian cells is invariably associated with production of immunogenic DNA waste triggering severe autoinflammation unless efficiently degraded.

## Material and Methods

### Mice

For reasons of clarity, we collectively refer to both, the *Trex1* knock out line published by Morita et al. (23) and the *Trex1^Δ478/Δ478^* line generated in this study, as *‘Trex1^-/-^’*. Trex1^Δ478/Δ478^ mice, which phenocopy the published line, were used in experiments of **Figure 2**, and **Figure S2**. *Trex1^Δ478/Δ478^Xpa^Δ11/Δ11^* mice were generated by microinjection of a mixture of four individual Cas9 RNPs into zygotes of C57BL/6 mice allowing simultaneous targeting of *Trex1* (GTGACTTCGGGCCGAGACGA and CGGTGCTTGCCAGTACAGGC) and *Xpa* (GTGTTTATCATCAGCATCTC and GAACCCACGCCATTCACAGTG). The injection mix contained 1.73 µM of each crRNA (IDT), 6.9 µM tracrRNA (IDT) and 6.9 µM Cas9 protein (ToolGen). *Trex1^FL/FL^ Rosa26-CreERT2^WT/KI^* mice were described earlier (25). Mice were housed under specific pathogen-free conditions at the Experimental Center, Medical Faculty Carl Gustav Carus, TU Dresden. All procedures were in accordance with institutional guidelines on animal welfare and were approved by the Landesdirektion Dresden (permit number TVV-88/2017).

### Induced Inactivation of *Trex1* in Primary Cells *In Vitro*

Bone marrow cells from *Trex1^FL/FL^R26^CreERT2^* or *Trex1^WT/FL^ R26^CreERT2^* control mice were cultured overnight in RPMI 1640 medium supplemented with 10% heat-inactivated FCS, 1% Pen/Strep, 2 mM L-Alanyl-L-glutamine and 1 mM sodium pyruvate (all Biochrom). Non-adherent cells were transferred to 10 cm petri dishes and differentiated for six days in RPMI medium (supplemented as described) containing 30 % L929 supernatant. Fresh differentiation medium was added after three days. BMDMs were harvested by trypsinization and scraping. For experiments addressing the kinetics of loss of TREX1, 2.5x10^5^ cells were seeded onto 6-well plates and treated with 2 µg/ml (Z)-4-Hydroxytamoxifen (Sigma, Cat#H7904) for 22 h. To address effects of cell cycle arrest on the IFN response after induced knock out of *Trex1*, 2x10^6^ cells were seeded onto 10 cm petri dishes and induced with 4-OHT. One day after washing away 4-OHT, cells from 10 cm petri dishes were split to 6 well plates at a density of 2.5x10^5^ and maintained in the presence of 5 µM Palbociclib (Selleckchem, Cat#S1116) for 72 h. Efficiency of Palbociclib-induced cell cycle arrest was verified by pulsing with 10 µM EdU for 4h at 37°C. Upon harvest, incorporated EdU was quantified flow cytometrically using the Click-iT™ EdU Alexa Fluor™ 647 Flow Cytometry Assay Kit (Invitrogen, Cat#C10419) following the manufacturer’s instructions.

MEFs were generated by standard procedures. Briefly, after caesarean section, E14.5 mouse embryos were decapitated and internal organs were removed. Tissue was cut into small pieces, digested with 1x trypsin (0.25%, Invitrogen) for 30 min at 37°C and disaggregated by pipetting. The cell suspension was seeded into 10 cm petri dishes with DMEM (Gibco) supplemented with 10% heat inactivated FCS, 1% Pen/Strep, 1x nonessential amino acids and 100 µM β-mercaptoethanol. Induction of Cre activity was done as described above. To address effects of cell cycle inhibition on the IFN response after induced knock out of *Trex1*, 1.1x10^5^ cells were seeded onto 6 cm petri dishes and induced with 4-OHT. On the next day, 4-OHT-containing medium was removed and fresh supplemented DMEM containing 200µM L-Mimosine (Sigma-Aldrich, Cat# M0253) was added to the cells for four days.

### Quantitative RT-PCR

Total RNA was isolated using NucleoSpin RNA Kit (Macherey-Nagel) and reverse transcribed into cDNA using PrimeScript RT Reagent Kit (Takara). qRT-PCR was performed using Luna^®^ Universal qPCR Master Mix (New England BioLabs) on a CFX384 Touch Real-Time PCR Detection System (Bio-Rad). Primer Sequences are listed in **Supplementary Table S1**. Target gene expression was normalized to the housekeeping gene *Tbp1*.

### Quantification of Micronucleated Erythrocytes

Blood was sampled using retrobulbar puncture and mixed with heparin/PBS (250 units/ml, Biochrom). 20 µl of the blood/heparin/PBS mixture was added to 2 ml ice-cold Methanol, inverted and stored at -80°C until further use. For quantification of micronucleated erythrocytes, the protocol published by Balmus et al. was followed (42). Briefly, fixed blood cells were washed in bicarbonate buffer and stained against CD71 and Ter119 in the presence of RNase A. After washing and addition of PI, cells were analysed by flow cytometry and gated for single Ter119^+^ CD71^-^ PI^+^ micronucleated normochromatic erythrocytes (MN-NCE). 5x10^5^ cells in P1 SSC/FSC gate were acquired.

### Assay for Nucleotide Excision Repair Activity

To detect ‘unscheduled’ DNA synthesis occurring in non-S phase cells repairing UV-induced genome damage (‘repair synthesis’), 5x10^6^ splenocytes from *Xpa^WT/WT^* and *Xpa^KO/KO^* mice were seeded onto 10 cm petri dishes in 3 ml PBS and irradiated with 20 J/m² UVC (xy min) using an OSRAM HNS 15W OFR 3224 lamp. 6 ml of serum-free RPMI containing 1% Pen/Strep, 1x NEA and 10 µM EdU was added for 3h. Cells were harvested and incorporated EdU was quantified flow cytometrically using the Click-iT™ EdU Alexa Fluor™ 647 Flow Cytometry Assay Kit (Invitrogen, Cat#C10419) following the manufacturer’s instructions.

### Western Blot Analysis

Ears of *Xpa^WT/WT^* and *Xpa^KO/KO^* animals were homogenized and lysed in RIPA buffer [50 mM Tris HCl, 150 mM NaCl, 1% NP-40, 0,1% SDS, 0,5% sodium deoxycholate, 1x Complete protease inhibitor (Roche)] for 30 min on ice. Lysates were cleared by centrifugation (16.000 xg, 10 min, 4°C) and supernatant was incubated with 6x Laemmli sample buffer at 95°C for 5 min. Cell pellets were directly lysed in 2x Laemmli sample buffer and incubated at 95°C for 5 min. Proteins were separated on 12% SDS polyacrylamide gels and transferred to a nitrocellulose membrane (Amersham Hybond-ECL, GE Healthcare). Membranes were blocked in 5% low fat dried milk/TBS-T or 1x Roti^®^Block and incubated overnight with the respective primary antibody. After washing in TBS-T, membranes were incubated with secondary antibodies for 1 h at room temperature and washed again in TBS-T. For signal detection, membranes were incubated in ECL (Amersham) or AP buffer following incubation in NBT/BCIP substrate (Roche).

The following primary antibodies were used: β-Tubulin (9F3, Cell Signaling Technology, Cat#2128), β-actin (13E5, Cell Signaling Technology, Cat#4970), Xpa (abcam, Cat#ab58464, 1:500), mTrex1 (C-11, Santa Cruz, Cat#sc-133112, 1:250), hTrex1 (E-6, Santa Cruz, Cat#sc-271870, 1:250), Mus81 (MTA30 2G10/3, Santa Cruz, Cat#58382, 1:250). Secondary antibodies used were AP-coupled anti-rabbit (Dako) and anti-mouse (Cell Signaling Technology).

### Histology

Hearts were fixed in 4 % formalin and embedded in paraffin. 3 µm paraffin sections were stained with hematoxylin and eosin. Inflammation was quantified by an experienced histopathologist in a blinded fashion. A histological score for H&E staining was given depending on the grade of inflammation (0 – no sign of inflammation, 1 - slight diffuse increase in inflammatory cell infiltration, 2 - few small dense focal inflammatory infiltrates, 3 - large inflammatory infiltrates extending through more than half of the thickness of the ventricular muscle plus focal destruction of myocardial structure). To judge the effect of additional loss of EXO1 on the intensity of myocarditis of *Trex1^-/-^* mice, density of T cell infiltration on sections stained for CD3 and tissue fibrosis on Masson’s trichrome-stained sections were analyzed. CD3 immunohistochemical staining was performed on a Ventana Discovery Ultra Instrument. In brief, antigen retrieval using cell conditioning 1 solution (Ventana Medical Systmes) was performed at 95°C for 32 min followed by incubation with anti-CD3 antibody (SP7, abcam, Cat#ab16669, 1:200) at 36°C for 32 min, anti-rabbit OmniMap HRP-coupled secondary antibody (Ventana Medical Systems) for 12 min and finally Opal 650 TSA fluorophore (Akoya Biosciences, 1:100) at RT for 8 min. Primary and secondary antibodies were removed by denaturation at 100°C for 24 min in cell conditioning 2 buffer (Ventana Mediacl Systems) before counterstaining with DAPI. Sections were scanned (100x magnification) and images aquired (x200 magnification) by Vectra 3.0 Automated Imaging System (Akoya Biosciences). CD3^+^ cells were quantified using inForm (Akoya Biosciences) and R software. Masson’s trichrome staining was done according to standard protocols (Sigma-Aldrich, Procedure No.HT15).

### Transcriptome Analysis

5 ng/µl RNA were sequenced on Illumina NovaSeq 6000 and data were demultiplexed using JE demultiplexer tool. Read quality was checked by FastQC. Transcript abundance was quantified using Salmon tool (88) and tximport (89) was used to generate gene level count matrices. The murine genome GRCm39 (90) was used as the reference. All post mapping analysis and visualization was conducted in R unless specifically stated otherwise. Normalization, exploratory analysis and differential expression analysis was performed using DESeq2 (91). Genes were considered significantly differentially expressed at FDR <0.01 and absolute log fold change >1.5. GGplot2 (92) was used to visualize results. List of significant DE genes was used to conduct a further gene set enrichment analysis (GSEA) (93, 94).

### Statistical Analysis

Unless stated otherwise, significance was calculated by One-way ANOVA with Tukey’s *post-hoc* test (**Figures 1**–**4**) or Two-way ANOVA with Sidak’s *post-hoc* test (**Figures 5**, **6**).

## Author’s Note

While our manuscript was under review, Kim et al. published that TREX1 indeed degrades oligonucleotide waste released upon UV irradiation by the nucleotide excision repair (NER) machinery (95), supporting our initial hypothesis that the IFN response of TREX1-deficient cells might be driven by NER-derived DNA waste. Such DNA might contribute to UV-induced inflammation, but not to STING activation in unirradiated TREX1-deficient cells, as we found no amelioration of the IFN response of *Trex1^-/-^Xpa^-/-^* mice compared to *Trex1^-/-^* mice.

## Data Availability Statement

The original contributions presented in the study are publicly available. This data can be found here: https://www.ncbi.nlm.nih.gov/search/all/?term=GSE197291.

## Ethics Statement

The animal study was reviewed and approved by Landesdirektion Dresden. Written informed consent was obtained from the owners for the participation of their animals in this study.

## Author Contributions

NS designed and performed experiments; TS, ED, and KF performed experiments; GK, LH, and YG analyzed transcriptome data; WE generated *Exo1* ^-/-^ mice; LM and MS performed and analyzed immunohistochemistry; VH and RB contributed to the design of the study and discussed data; AR conceived and supervised the study; NS and AR wrote the manuscript. All authors contributed to the article and approved the submitted version.

## Funding

This work was supported by DFG grants Collaborative Research Center 237 Nucleic Acid Immunity, project B17 to AR, project B19 to RB and project A09 to VH, MedDrive Grant 60.496 of the Faculty of Medicine Carl Gustav Carus, TU Dresden to NS.

## Conflict of Interest

The authors declare that the research was conducted in the absence of any commercial or financial relationships that could be construed as a potential conflict of interest.

## Publisher’s Note

All claims expressed in this article are solely those of the authors and do not necessarily represent those of their affiliated organizations, or those of the publisher, the editors and the reviewers. Any product that may be evaluated in this article, or claim that may be made by its manufacturer, is not guaranteed or endorsed by the publisher.
