# Supplementary material for: Genome Replication Is Associated With Release of Immunogenic DNA Waste
Source: Front Immunol. 2022 May 11;13:880413. doi: 10.3389/fimmu.2022.880413 (PMC9130835; doi:10.3389/fimmu.2022.880413)
Supplement: Supplementary file 1 [file DataSheet_1.docx]

Supplementary Material

**Supplementary Table 1. Primer sequences for qPCR (5’-3’).**

| **Gene** | **Forward Primer** | **Reverse Primer** |
| --- | --- | --- |
| mTbp1 | TCTACCGTGAATCTTGGCTGTAAA | TTCTCATGATGACTGCAGCAAA |
| mIfi44 | GGCACATCTTAAAGGGCCACACTC | CTGTCCTTCAGCAGTGGGTCATG |
| mOasl1 | CGTTGTGCCCGCCTACAGAGCC | GCTGCAGCTCGCTGAAGGATGG |
| mUsp18 | CACAACATCGGACAGACGTGTTGC | CTTCCTCTCTTCTGCACTCCGAG |
| mIsg15 | TAGAGCCTGCAGCAATGGCCT | GGAAAGCCGGCACACCAATCT |
| mOas1a | GCTGCCAGCCTTTGATGT | TGGCATAGATTCTGGGATCA |
| mIfit3 | TGAACTGCTCAGCCCACA | TCCCGGTTGACCTCACTC |
| mIrf7 | GTGTACGAACTTAGCCGGGA | GCATTGCTGAGGCTCACTTC |
| mCxcl10 | GCCGTCATTTTCTGCCTCAT | GCTTCCCTATGGCCCTCATT |
| hHprt | TGCTGAGGATTTGGAAAGGG | GCTACAATGTGATGGCCTCC |
| hIfi44 | TTTCCAAGGGCATGTAACGC | TTTGCCATCTTTCCCGTCTC |
| hIfi27 | ACCAAGTTCATCCTGGGCTC | TGGTTCTCTTCTCTGCAGGG |
| hIfit1 | CCTGGCTAAGCAAAACCCTG | CAGATGGCTCCTTCTTGCTG |
| hIsg15 | TTTGCCAGTACAGGAGCTTG | GGGACACCTGGAATTCGTTG |
| hCxcl10 | GCAAGCCAATTTTGTCCACG | TTGATGGCCTTCGATTCTGG |


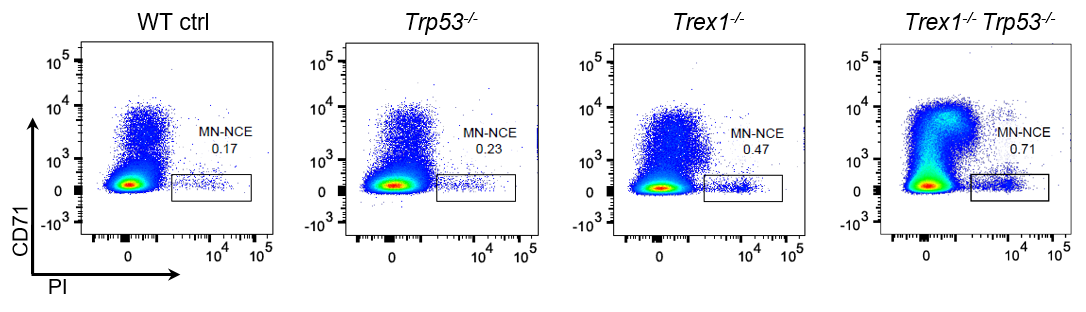


**Supplementary Figure 1. Flow cytometric quantification of micronucleated erythrocytes.** Representative result of flow cytometric analysis of Ter119^+^ fixed blood cells for quantification of micronucleated normochromatic erythrocytes (MN-NCE) of WT, *Trp53^-/-^*, *Trex1^-/-^*, and *Trex1^-/-^ Trp53^‑/-^* mice as summarized in Fig. 1a.


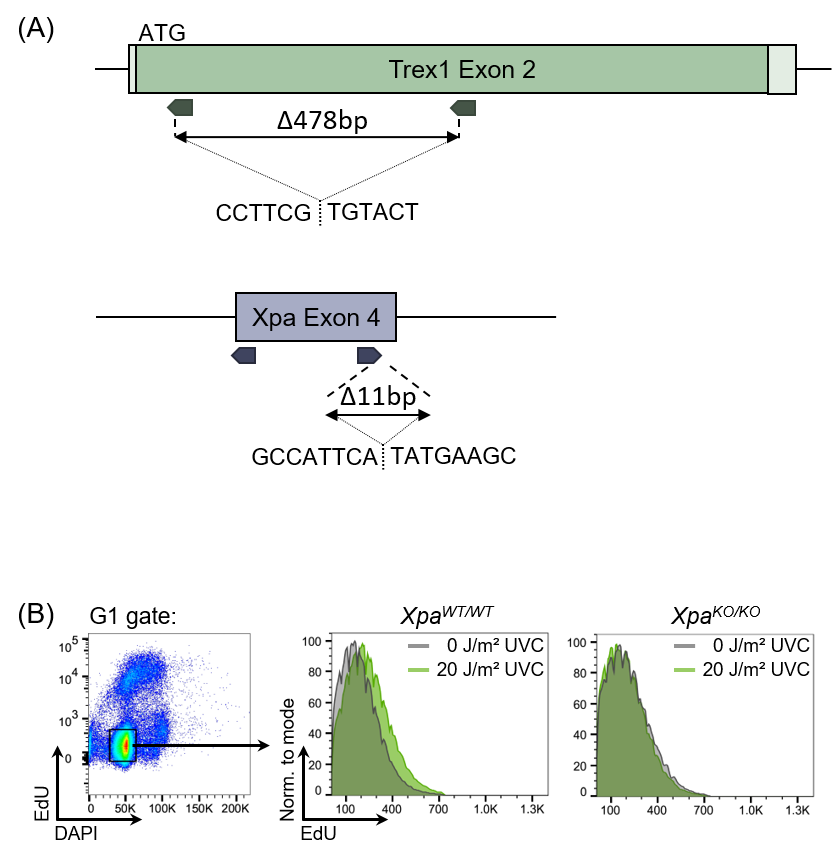


**Figure S2. Simultaneous inactivation of the essential nucleotide excision repair gene *Xpa* and *Trex1* in vivo.**

A) Schematic representation of the deletions obtained by CRISPR/Cas9-mediated gene targeting in C57BL/6 embryos. Positions of guides are indicated as green or purple arrows. The 478bp (out of frame) deletion eliminates the upstream half of the *Trex1* coding region. Mice homozygous for the new *Trex1^Δ478^* mutation are phenotypically indistinguishable from the *Trex1^-/-^* strain published by Morita et al. (Morita et al., 2004). The 11bp deletion in the *Xpa* gene, encoding an essential NER protein, introduces a frame shift into exon 4.

B) Loss of NER functional activity in mice homozygous for the new *Xpa^Δ11^* allele (‘*Xpa^KO/KO^’*). In wild type cells, repair of UV-induced damage involves excision of lesional DNA as a short oligonucleotide and subsequent resynthesis which can be detected by EdU incorporation as ‘unscheduled DNA synthesis’ in non-S phase cells (Limsirichaikul et al., 2009). Total splenocytes were exposed to UVC *in vitro* in the presence of EdU followed by flow cytometric quantification of EdU incorporation in G1 cells. Left: gating of G1 cells. Right: increase in EdU staining upon UV irradiation in *XPA^WT/WT^* cells, but not in *XPA^-/-^*cells. FACS plots representative of 3 mice per genotype.

****p< 0.0001, ***p<0.001, n.s. not significant.


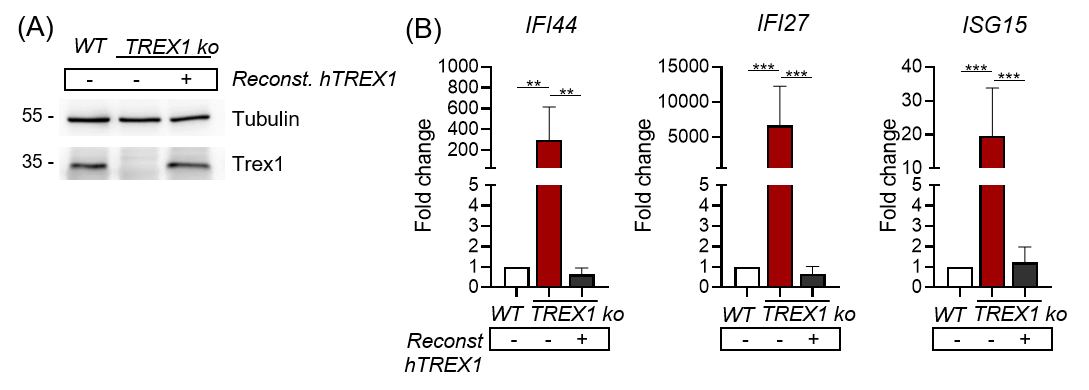


**Figure S3**. **Loss of Trex1 expression results in spontaneous IFN signaling in THP1 cells.**

A) Inactivation of *Trex1* in THP1 cells by CRISPR/Cas9-mediated gene targeting. Mixtures of pooled TREX1-deficient clones were used for subsequent experiments. Reconstitution with functional *Trex1* by lentiviral gene transfer results in re-expression of TREX1.

B) ISG transcript levels in parental WT THP1 cells and in the pool of TREX1-deficient clones quantified by qRT-PCR. Fold change compared with mean of levels in the parental cells is shown. Reconstitution of TREX1 expression (5 reconstituted clones were mixed) reduces ISG transcripts to control levels.

***p<0.001, **p<0.01.


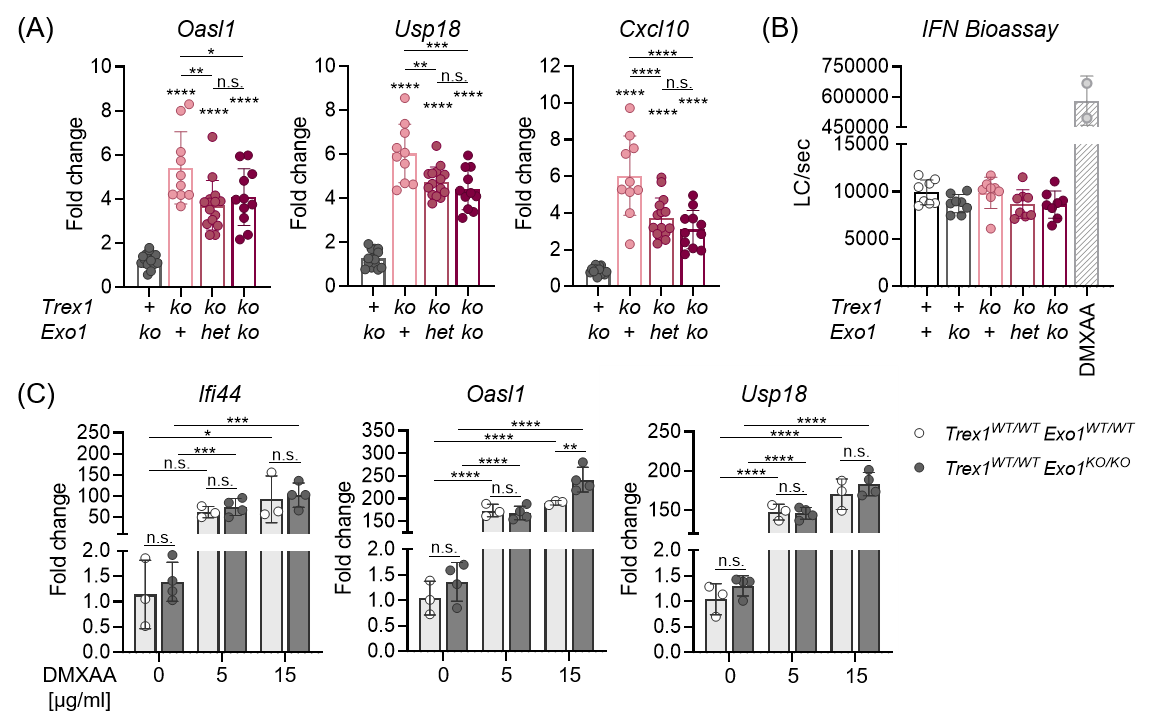


**Figure S4. Additional loss of EXO1 ameliorates IFN signalling in TREX1-deficient cells despite normal responsiveness of the cGAS/STING axis in *Exo1*^-/-^ cells.**

A) ISG transcript levels in total blood cells from 6 week-old mice of the indicated genotypes quantified by qRT-PCR. Fold change compared with mean of WT controls (n=15) is shown (*Trex1^-/-^* n=10, *Exo^‑/-^* n=14, *Trex1^-/-^Exo1^+/-^* n=15, *Trex1^-/-^Exo1^-/-^* n=11).

B) Assay for type I IFN bioactivity in sera of 6 week-old mice of the indicated genotypes. 1:10 diluted sera were added to 1x10^4^ LL171 luciferase IFN-reporter cells for 6h followed by quantification of luciferase activity (n=8 per genotype). DMXAA-treated LL171 cells were used as positive control.

C) Baseline and DMXAA-induced IFN signalling of *Exo1^-/-^* splenocytes as determined by qRT-PCR-based quantification of ISG transcript levels, compared to levels of wildtype cells which were set to 1.

***p<0.001, **p<0.01, n.s. not significant.


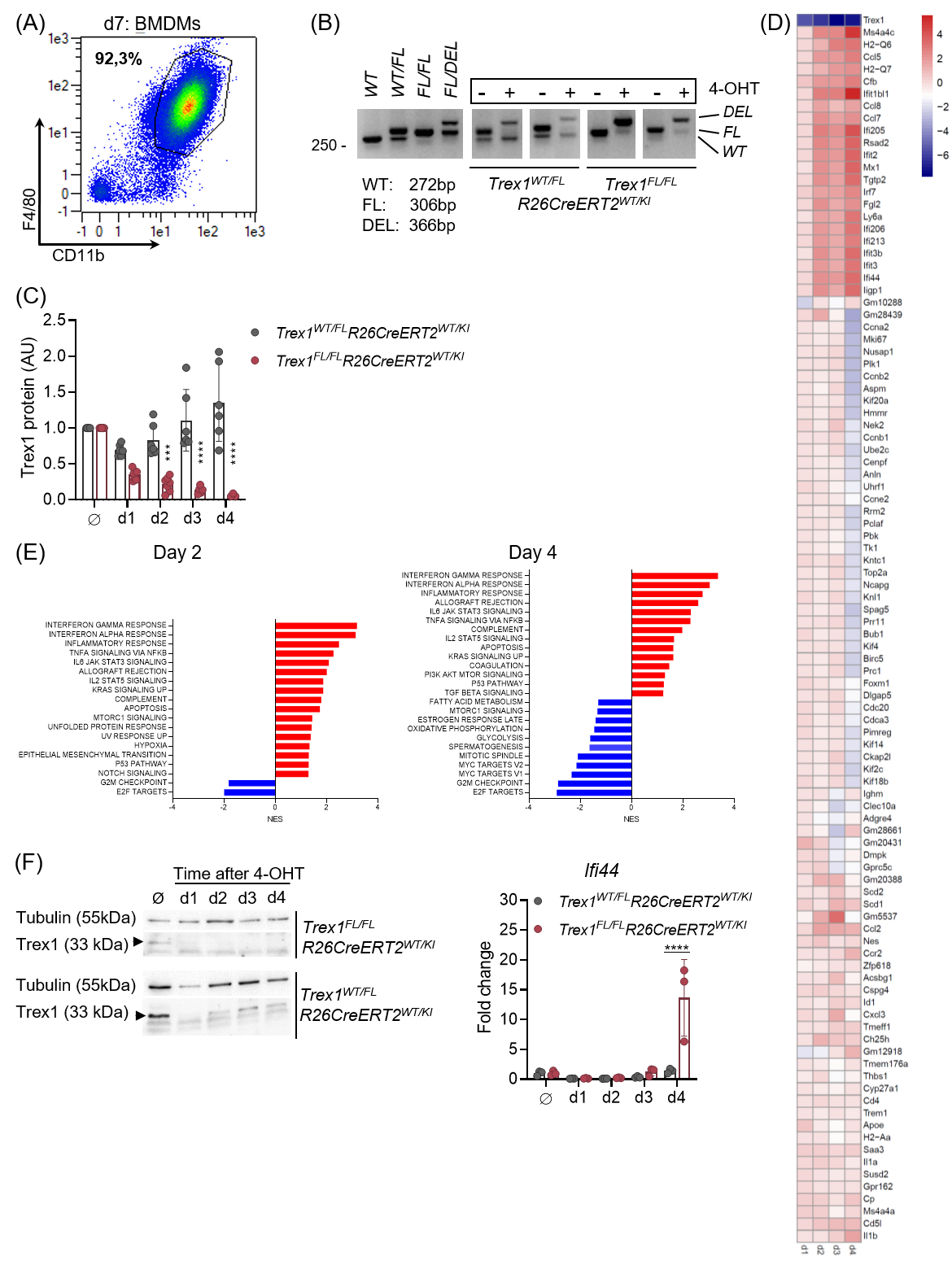


**Figure S5. Rapid activation of IFN signalling upon induced inactivation of Trex1 in macrophages and fibroblasts *in vitro*.**

(A) Representative FACS analysis of BMDMs differentiated from bone marrow for 7 days in L929-conditioned medium.

(B) PCR analysis of the *Trex1* locus from *Trex1^FL/FL^R26^CreERT^*or *Trex1^WT/FL^R26^CreERT2^* BMDMs 4 days after 4-OHT induction. Left, results for standard DNA samples from cells of the indicated genotypes. Right, DNA from 4-OHT-treated *Trex1^FL/FL^R26^CreERT2^*and control cells.

(C) Densitometric evaluation of TREX1 protein levels on Western blots of 4-OHT-induced BMDMs (*Trex1^FL/FL^R26^CreERT2^*, n=7; and control cells, n=6) as in Fig. 5B.

(D) Heatmap of the most deregulated genes compared to mean of control cells from the same timepoint.

(E) Gene sets enriched at in 4-OHT-induced *Trex1^FL/FL^R26^CreERT2^*BMDMs (red) or control cells (blue) with a false discovery rate below 0.01.

(F) Left: Western blot analysis of TREX1 expression in MEFs at the indicated time points after 4-OHT induction, representative of 3 animals per genotype. Right: Transcript levels of the ISG *Ifi44* determined by qRT-PCR in MEFs sampled at the indicated time points after 4-OHT induction. Fold change compared to untreated control cells (n=3) is shown.

****p< 0.0001, ***p<0.001.


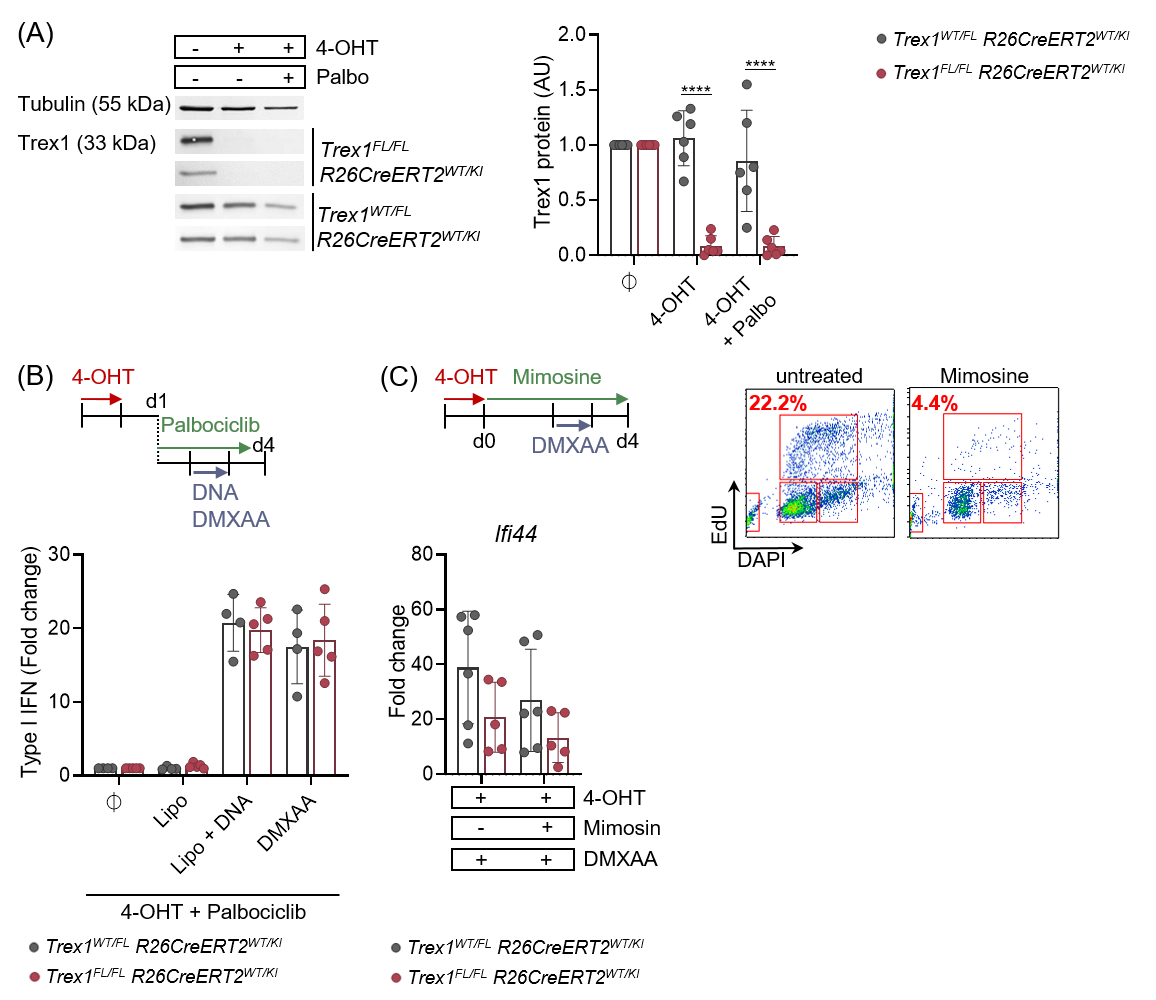


Figure S6. IFN response upon induced inactivation of Trex1 requires ongoing proliferation

(A) Western blot analysis of TREX1 expression in BMDMs treated with 4-OHT and Palbociclib (right) and densitometric evaluation of TREX1 protein levels (left; n=6 per genotype). Samples were obtained on day 4 after end of 4-OHT induction.

(B) 4-OHT-induced *Trex1^FL/FL^R26^CreERT2^* and control (*Trex1^WT/FL^R26^CreERT2^*) BMDMs were transfected with DNA (for 2h) or treated with DMXAA (for 24h) while arrested with Palbociclib. Type I IFN bioactivity was assayed in the supernatant as described before (n=4 per genotype, pooled from 2 independent experiments).

(C) 4-OHT-induced *Trex1^FL/FL^R26^CreERT2^* and control (*Trex1^WT/FL^R26^CreERT2^*) MEFs were treated with DMXAA (for 24h) while arrested with Mimosine. Transcript levels of the ISG *Ifi44* were determined by qRT-PCR (n=6 per genotype, pooled from 2 independent experiments). Fold change compared to mean of untreated control cells (n=6) is shown. FACS plots show proliferative arrest after 96h of Mimosine treatment as determined by quantification of EdU incorporation and DNA content (DAPI). Percentages indicate fractions of cells in S-phase.

****p< 0.0001.
